# Bridging the chasm between patients’ needs and current rehabilitation care: perceptions of adults presenting for primary care in the Eastern Cape

**DOI:** 10.1186/s12913-024-10564-5

**Published:** 2024-02-05

**Authors:** Maria Yvonne Charumbira, Thandi Conradie, Karina Berner, Quinette Abegail Louw

**Affiliations:** Division of Physiotherapy, Department of Health and Rehabilitation Sciences, Faculty of Medicine and Health Sciences, Stellenbosch University, Cape Town, 7500 South Africa

**Keywords:** Primary Health Care, Rehabilitation, Functioning problems, Access

## Abstract

**Background:**

The need for rehabilitation in low-to-middle income countries (LMICs) is rapidly increasing as more people are living longer with chronic diseases. Primary health care (PHC) is ideally placed to provide the spectrum of care required to meet most of the complex and evolving population's health needs locally. This study aimed to describe the patient journeys of adults attending primary care in the Eastern Cape province of South Africa to understand the factors that affected their access to primary care rehabilitation services (or the lack thereof) and obtain suggestions on how rehabilitation may be enhanced at primary care.

**Methods:**

A maximum variation sampling approach was used to purposefully select persons with varied chronic health conditions and demographic characteristics to gain diverse perspectives regarding their rehabilitation needs and ways in which the current rehabilitation services at primary care may be enhanced. Data were collected via face-to-face semi-structured interviews between March and June 2022 which were electronically recorded. Inductive thematic analysis of transcribed data was done and coded in Atlas.ti.22®.

**Results:**

Twenty-five adult patients participated in the study. The patients had different experiences at their local PHC facilities that affected their access to rehabilitation at primary care. The study found that most patients were not able to access rehabilitation at primary care. There were several personal and contextual factors that resulted in the patients having a low perceived need to receive rehabilitation that potentially lowered patients' demand for and utilization of rehabilitation at primary care. Patients suggested increasing rehabilitation workforce at primary care, improving availability of assistive devices, increasing their knowledge regarding rehabilitation, and facilitating socio-economic integration into their communities.

**Conclusions:**

Patients attending primary care are not guaranteed access to rehabilitation by virtue of having entered the PHC system. It is important to consider the patient perspectives regarding their health needs and suggestions for enhancing care.

**Supplementary Information:**

The online version contains supplementary material available at 10.1186/s12913-024-10564-5.

## Introduction

The need for rehabilitation in low-to-middle income countries (LMICs) is already large and rapidly growing [1]. The increasing prevalence of better pharmacologically managed chronic disease in LMICs, along with ageing populations, points to a need for healthcare systems to better meet the rehabilitation needs of people living longer with more complex health needs [2, 3]. The need is more significant in South Africa's healthcare system, which must cope with the quadruple burden of disease arising from communicable diseases such as HIV and Tuberculosis (TB), maternal and child mortality, injury and trauma, and non-communicable disease (NCDs) [4]. The health conditions contributing to greatest disease burden in South Africa are associated with significant functioning problems related to mobility, pain and mental health [5].

Primary health care (PHC) is ideally placed to provide the spectrum of care required to meet most of the complex and evolving population’s health needs locally [6]. However, South Africa’s persistent economic divide negatively impacts healthcare provision. Approximately 16% of South Africans receive private healthcare while about 84% of mostly poorer populations utilize public healthcare [7]. The Eastern Cape has remained the poorest South African province since 2001, [8] with 77.2% of its population living below the poverty datum line compared to the national average of 55.5% in 2015, [9] and having poor access to quality healthcare services [10]. To redress these disparities, South Africa is implementing a national health insurance (NHI) system to ensure universal access to appropriate and quality health services, [7] with PHC as the ‘heartbeat of the NHI.’ [6] Additionally, South Africa’s National Rehabilitation Policy: 2000 seeks to restructure rehabilitation services to improve access for people suffering from health conditions that can lead to disability as well as those living with disabilities [11]. More recently, the Framework and Strategy for Disability and Rehabilitation Services in South Africa (FSDR): 2015 – 2020 aimed to provide comprehensive rehabilitation services at each level of care based on community-based rehabilitation principles, which emphasise inclusion and integration of persons into their communities [12].

Rehabilitation forms one of the five pillars of primary care [13] and must be integrated into PHC to effectively meet the needs of the population. Currently there is a shortage of rehabilitation professionals at primary care, especially in rural areas [14]. Access to rehabilitation services in PHC is often contingent on initial consultation and referral by primary care providers (PCPs) at Community Healthcare Centers or Clinics, who are often nurses or doctors. At times, mobile rehabilitation teams from the district hospitals provide outreach rehabilitation services to rural or remote clinics. However, patients may have to seek care at higher levels of the healthcare system if rehabilitation services are not available leading to resource burden and increased costs [15]. Utilization of rehabilitation at primary care settings can be more effective when services are provided nearer to the home and work settings as it allows closer integration with patients’ daily activities [15] and promotes self-management of identified functioning problems within the patients’ environment [16]. Continued follow-up at primary care for patients discharged from higher levels of care can facilitate recovery and optimise the effects of acute treatment interventions.

The poorer and more vulnerable populations are often excluded from health service planning and policy-making decisions and yet they are most affected by poor access to quality healthcare. Levesque et al. developed a conceptual framework that considers multiple dimensions of access to healthcare to understand disparities in healthcare access and utilization taking into account the health systems and patients’ perspectives [17]. Obtaining feed-back from the end-users’ experience of the PHC system will be useful in evaluating and identifying key areas for improvement that will enable health systems to respond to the populations’ specific needs in a meaningful way [18]. Moreover, the National Rehabilitation Policy advocates for participation of persons in need of rehabilitation, including people with disabilities, in the whole process of rehabilitation services and policy development and implementation [11].

No studies have been found that explored factors affecting PHC patients access to rehabilitation at primary care in low-resource settings. One South African study reported on patients’ perspectives or experiences of rehabilitation provided at an urban community health centre [19]. The study reported patients’ difficulties in accessing rehabilitation due to lack of transportation, limited resources, and insufficient information about rehabilitation services. Scheffler et al. explored an urban community populations’ access to PHC and briefly mentioned healthcare system-related challenges faced by people with disabilities in accessing rehabilitation [20]. These included lack of rehabilitation services at the PHC facilities (consisting of Community Healthcare Centres (CHCs) and day clinics), [21] inadequate referral, and long waiting times. However, none of the studies have focused on the challenges experienced by patients in both rural and urban areas, who have accessed PHC and may require rehabilitation, but have not been able to access rehabilitation. Furthermore, the studies have not sought to obtain patients’ suggestions on how rehabilitation services may be enhanced.

Health systems should address what matters to people about their health, that is, their “lived health”. This process of contextualization streamlines efforts and saves limited resources by prioritizing the rehabilitation needs that are of key concern to the community and shaping rehabilitation services that are specifically for them. Thus, it is crucial to incorporate the person-centred perspectives of persons with functioning problems in planning and delivering rehabilitation services [22]. Including the users of rehabilitation as stakeholders is particularly important for South Africa given the planned NHI and should be done from inception of the planned care packages. This study aimed to describe the patient journeys of adults attending primary care in the Eastern Cape province of South Africa to understand the factors that affected their access to primary care rehabilitation services (or the lack thereof). The study further sought to obtain their perceptions regarding how the current rehabilitation services at primary care may be enhanced.

## Methods

### Design

An exploratory, descriptive, qualitative study was used since little is known about this topic [23]. A phenomenological approach with an interpretive paradigm was used to understand how some adults attending primary care made sense of their need for rehabilitation at primary care [24]. The report followed the consolidated criteria for reporting qualitative research (COREQ) [25] and the standards for reporting qualitative research (SRQR) [26].

### Setting

**Fig. 1:**
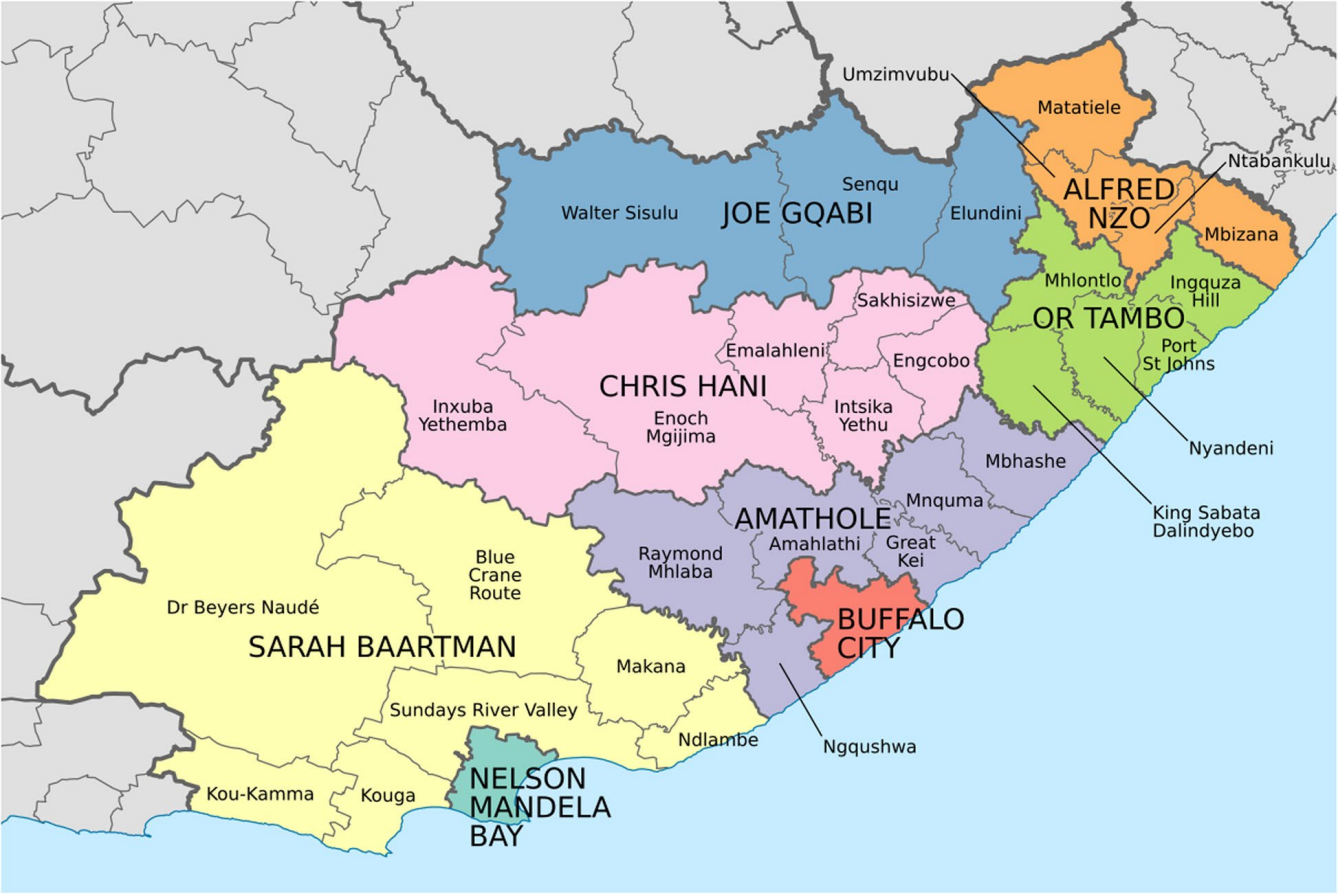
Map of the Eastern Cape with municipalities named and showing the rural and urban districts. In Wikipedia. https://commons.wikimedia.org/wiki/File:Map_of_the_Eastern_Cape_with_municipalities_named_and_districts_shaded_(2016).svg

The Eastern Cape is the second largest province of the nine provinces in South Africa. It has the fourth highest population which constitutes 11.1% of the country’s total population. About 80% of its inhabitants speak isiXhosa and 60% live in rural areas. The Eastern Cape province is divided into two metropolitan municipalities (Buffalo City and Nelson Mandela Bay) and six district municipalities (Alfred Nzo, Amathole, Chris Hani, Joe Nqabi, OR Tambo and Sarah Baartman) (Fig. 1). The most recent intercensal demographic survey (2017) showed that the Eastern Cape had the third highest disability prevalence in the country (4.9%) [27]. The Eastern Cape also had the highest percentage (41.8%) of grant beneficiaries in the country. A grant is government-funded social or financial assistance provided to individuals who are unable to work or become financially independent, including the elderly, children or people with disabilities [28]. In terms of healthcare, 71.2% of households utilized public clinics or hospitals as their first point of access when household members required medical attention, while 10.7% had medical aid insurance cover [27]. The Eastern Cape has a total of 41 CHCs and 727 clinics. The CHCs are typically larger PHC facilities and deliver more comprehensive 24-h services than at the day clinics [21]. These services at CHCs are provided by a more extensive team of healthcare professionals, including doctors, nurses, pharmacists, and allied health professionals compared to mostly nurses at day clinics [21].

### Study site selection

Multi-stage sampling techniques were used to select the study sites. Firstly, one metropolitan district (Buffalo City) and one rural district (Amathole) were pragmatically selected for feasibility purposes, considering time and travel costs. Next, quota sampling was used to choose the PHC facilities according to geographic location (urban, traditional, and rural) and provision of rehabilitation services (Table 1). Traditional settings refer to the smaller agricultural-based urban towns of rural or commercial farming settlements, which provide a different setting from the large metropolitan districts, that have higher population density and extensive infrastructure. Quota sampling is a non-probability sampling technique in which the researcher selects a sample from each of the already existing distinct groups in a population based on predetermined criteria or preference) [29].

**Table 1:** Quota sampling distribution of clinics according to geographic location and provision of rehabilitation

	**Urban**	**Traditional**	**Rural**
Rehabilitation	1	1	1
No Rehabilitation	1	1	1

### Study participants and recruitment

Adults (older than 18 years) seated in the waiting rooms of the selected PHC facilities and had agreed to participate in the study were included if they had a diagnosed health condition and self-reported that they had any functioning problems. Participants with cognitive, hearing, or verbal impairments which made it difficult to articulate their responses were included if they had a caregiver who could assist with obtaining responses. A caregiver was considered the person who accompanied the patient on the day of the research visit or spent the most time caring for the patient. A maximum variation sampling approach was used to purposefully select persons with varied health conditions and demographic characteristics to gain diverse perspectives regarding their rehabilitation needs and ways in which rehabilitation services may be enhanced.

According to recommended sample size for phenomenological studies, [23] it was proposed a priori that at least 4 – 5 participants per PHC facility (i.e., a total sample size of 24 – 30 participants) would be interviewed. However, purposive sampling prescribes continued sampling until data saturation is achieved until no new significant information is obtained [30]. Thus, patients continued to be sampled until data saturation was achieved.

### Instrumentation

Data collection tools included a brief self-reported socio-demographic and medical history questionnaire and a semi-structured interview guide (provided as Additional files 1 and 2). The semi-structured interview guides were developed according to similar qualitative studies that sought to explore patients’ access and quality of healthcare [19, 31].

### Data collection

Data were collected via 20 to 30-min-long face-to-face interviews between March and June 2022. The principal investigator conducted 23 interviews in isiXhosa with the assistance of a language interpreter and 2 interviews in English. A research assistant was present throughout all interviews to take brief notes and for quality assurance. The scheduled interview guide was followed to ensure that all themes of interest were covered. Further probing questions were used to clarify responses and gain a deeper understanding. No pilot interviews were conducted due to time constraints during the research visits. However, new relevant issues that arose during preceding interviews were addressed in subsequent interviews. In addition, one repeat interview was conducted to seek further clarity on issues that had not been asked during the initial interview. Member-checking was done throughout the interviews to check that the participants’ expressions were correctly understood by the researchers.

The interviewers and interviewees were seated in closed rooms at the PHC facilities, free of disruptions. Non-participants were not allowed into the rooms while interviews were conducted to ensure privacy and confidentiality, thus facilitating honest responses.

### Audio recordings

Interviews were recorded electronically after obtaining verbal consent from each participant. After allocating unique study IDs, electronic recordings were safely stored in a password-protected file on the PI's laptop.

### Data processing

The recordings were transcribed verbatim with the assistance of professional transcribers. Transcripts were returned to five participants via WhatsApp (a popular chat and instant messaging application) to check that transcribed accounts accurately reflected what they had said. Participants' names were removed from the transcripts. Transcripts continued to be identified by the same unique IDs allocated to the audio recordings.

### Data analysis

Analysis was an iterative process involving repeated cycles of data collection, transcription and analysis [30]. Thematic content analysis using an inductive approaches was applied [32]. The initial coding of the transcripts was conducted by one member of the research team (MC) by repeatedly reading transcripts to identify common conceptual themes and patterns that emerged from the data. Several discussions of the preliminary coding were done by all members of the research team (MC, TC, KB and QL) to share perspectives and understanding of participants accounts. Differences were discussed until consensus was reached. Thus, an iterative process of naming, renaming, and redefining codes, and identifying recurring themes, concepts and patterns and organising them into categories was followed. Member checking was done by returning transcripts to some participants to validate transcription and interpretation to ensure that participants agreed with the researchers’ interpretation of the emerging themes [33]. A codebook was created, which was applied to the rest of the transcripts with the aid of Computer Assisted Qualitative Data Analysis (CAQDAS) software, Atlas.ti. version 22.2®.

### Trustworthiness

Several strategies were used to ensure credibility, dependability, transferability, and confirmability [34]. Credibility was ensured by employing purposive sampling techniques and presenting the various viewpoints held by the participants. Data triangulation was done by consulting notes taken by interviewers as well as the reflexive notes recorded by the PI. Transferability was enabled through rich descriptions of the participants and the research methods used in this study. To ensure dependability, exemplary quotations from most participants were provided to support the emerging themes. Member checking ensured confirmability. An audit trail was provided through detailed documentation of the research process.

Qualitative research acknowledges that each researcher brings a unique perspective to the study. Reflexive analysis was done to improve the confirmability of the study by the primary investigator (PI), acknowledging any influence or personal biases that may have affected the study results. The PI acknowledged her limitations in not being familiar with the culture and language of the Eastern Cape. As a result, she may have missed some of the meanings ascribed to participants' accounts. However, there could be an advantage in an element of objectivity from setting herself apart as an observer. The PI was the main interviewer, although a second interviewer (TC) was present to ensure consistency and coherence throughout the interview process. The researcher documented her continual reflections on the research process, including her thoughts, actions, and decisions, in a reflexive journal.

### Ethical considerations

Ethical approval was obtained from the Health Research Ethics Committee of Stellenbosch University (S21/01/002 (PhD)), and the Eastern Cape Department of Health and PHC facility managers granted permission to conduct the study. Written informed consent was obtained from each participant before the interviews. Written informed consent was obtained from the legal guardians of participants with no education or with cognitive or mental impairments.

## Results

### Participant characteristics

A total of 28 adult participants were invited to participate in this study. Two participants declined participation due to fear of not being able to provide enough information on the topic, while one declined due to lack of time. Thus, 25 interviews were conducted during the two week-long research visits. Two of the participants had caregivers who sat in on the interviews. The PI did not have to recruit more participants because data saturation was achieved. Table 2 summarises the participants' sociodemographic characteristics.

**Table 2:** Sociodemographic and clinical characteristics of the sample participants

Total participants	*n* = 25
Average age (range)	52.4 years (20 – 74)
Married	40%
Education	
*None*	12%
*Below Grade 12/ Standard 10*	72%
*Grade 12/ Standard 10 and above*	16%
Employed	25%
Source of income	
*Salary/wages*	24%
*Pension*	28%
*Grant*	40%
*None*	8%
Chronic health conditions (top five)	
More than one chronic health condition	76%
*HIV (93% on ART)*	56%
*Fractures (old)*	28%
*Tuberculosis*	20%
*Backpain*	20%
*Stroke*	16%
Used assistive devices (wheelchairs, crutches, walking sticks)	32%
Received rehabilitation at primary care	
*Yes*	12%
*No*	88%

*ART* Antiretroviral therapy, *HIV* Human Immuno-deficiency Virus

### Main findings

The mapping of data on barriers and facilitators affecting access to rehabilitation and recommendations for improving primary care rehabilitation was done following the main identified themes, subthemes and categories presented in Table 3. Some patients will be accessing the PHC system for the first time while others are re-accessing for follow-up visits but still experience the same barriers and facilitators.

**Table 3:** Themes, subthemes, and categories that arose from the participants’ responses

	**Theme**	**Subthemes**	**Categories**
1	Descriptions of patient journeys	Experiences of accessing PHC	
		Experiences at PHC	Reporting functioning problems to PCPs
			Referral to rehabilitation at PHC
		Outcomes of PHC visit	
2	Perceptions of current primary care rehabilitation		
3	Suggestions from patients for enhancing current primary care rehabilitation		

*PCP* Primary care providers, *PHC* Primary Health Care

### Descriptions of patients’ journeys

**Fig. 2:**
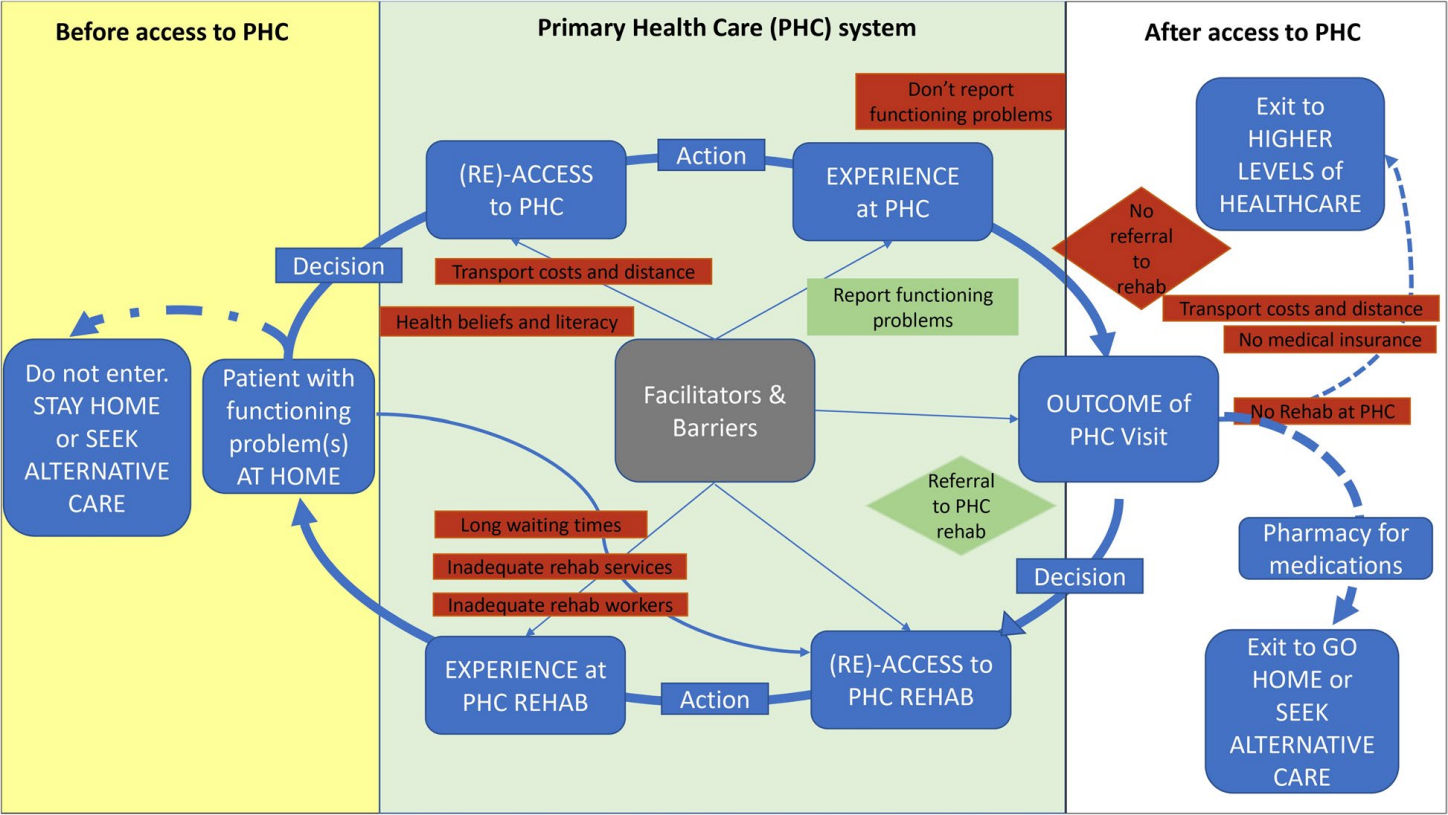
Summary of the different patient journeys of patients attending PHC leading up to the experience of rehabilitation at PHC or lack thereof. *(Re)- access refers to either accessing or re-accessing for subsequent visits

The participants’ responses indicated that the patients had different experiences at their local PHC facilities that affected their access to rehabilitation at primary care. Throughout the journeys (summarised in Fig. 2), patients were faced with different factors that hindered (barriers) or enabled (facilitators) their ability to perceive need, seek, reach, pay, and utilise rehabilitation services at different points of access within the PHC system. The descriptions of the patients’ journeys began with factors that affected their decision to leave home (highlighted in yellow), access and experience primary care, among which primary care rehabilitation was one of the services nested (highlighted in green). Most patients were not able to access rehabilitation at their PHC facility and experienced different outcomes of their PHC visit (highlighted in white). The few patients who managed to receive rehabilitation at primary care reported on their perceptions of the rehabilitation services and factors that affected their re-access for further rehabilitation. The detailed descriptions of these patient journeys are provided with exemplary quotations to support the narrated findings.

### Experiences of accessing PHC

In the study, most patients attended PHC primarily to collect their chronic medications. Thus, though the selected patients experienced functioning problems, these were not the reason for their visit. However, the participants demonstrated their capabilities in overcoming several barriers to access PHC including health beliefs, incurred costs, and physical distance.

The health beliefs of the patients or their families at large affected the patients’ ability to perceive their need for healthcare. For example, two participants reported overcoming pressure from family to seek alternative treatment from traditional healers instead of medical treatment. After medical treatment is deemed ineffective, common African traditional beliefs may lead family to ascribe functioning problems to spiritual or supernatural causes, thus, seeking further medical treatment would be regarded as futile.

“They suggested that I go see a Traditional healer. Because there was nothing helping me. But my family does not believe in that… I prefer hospitals and I prefer tablets over traditional medicine.” (P13, rural PHC facility).

These patients had sufficient health literacy to understand the need for them to seek medical healthcare and adhere to their treatment but may not have been knowledgeable about their rehabilitation needs.

The patients also overcame barriers to their physical access to the PHC facilities including transport costs and physical distance. Although the patients did not have to pay for the actual healthcare, indirect costs related to healthcare included transport costs. As a result of her difficulty with walking, one participant described having to pay for transportation even when the distance to the PHC was walkable.

“It's only the fact that I struggle to walk, so I'm forced to take transport and sometimes when I don't have money, I have to go borrow money for transport.” (P10, rural PHC facility).

The patients’ economic capacity and willingness to pay for the indirect costs was affected by the availability of social assistance in the form of disability grants, social grant and workers’ compensation for injuries sustained at work. Patients who were injured at work but were not compensated resented having to pay for their own healthcare expenses. Some participants relied on social grant money for individual or family sustenance but as one participant explained that they *“have to always borrow money to go see doctors. Because the social grant money does not cover everything.”* (P21, traditional PHC facility).

The barriers related to costs and distance may persist in impeding patients’ access to rehabilitation, particularly when rehabilitation services are not provided at the PHC facilities that they attended and additional costs are incurred in seeking this care elsewhere.

### Experiences at PHC

**Fig. 3:**
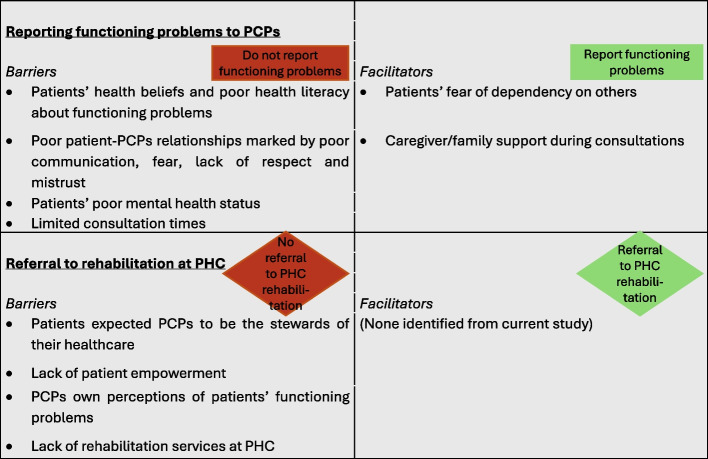
Barriers and facilitators to reporting their functioning problems to PCPs

The participants described their varied experiences at PHC which determined the outcome of their visit. Patients reported several factors that affected their ability to report their functioning problems to the PCPs and their subsequent referral to rehabilitation (Fig. 3).

#### Reporting functioning problems to PCPs

The first category arising from the participants’ experiences at PHC related to reporting their functioning problems. Several factors hindered patients from raising concerns related to their functioning problems when consulting with their PCPs:

##### Patients’ health beliefs and poor health literacy about their functioning problems

Some health beliefs resulted in patients not perceiving the need to address their functioning problems. Functioning problems were believed to be inevitable or ‘normal’ outcomes attributed to the diagnosed health condition or aging. For example, a 53-year-old patient felt that her low energy level “*has got something to do with [her] age”* (P14, rural PHC facility), while another patient who *“had been diagnosed with HIV… felt like [her backpain] was because of this status”* (P7, urban PHC facility). Therefore, they felt there was no need to catastrophize their experiences as the functioning problems were viewed as typical or expected.

The frequency with which they experienced the functioning problems influenced whether the participants perceived the need to report their problems to the PCPS. They were less likely to report if the functioning problems *“only happened once”* (P6, urban PHC facility) or do not *“experience all the time.”* (P11, rural PHC facility).

The above health beliefs suggested that the patients had poor health literacy and awareness of their rehabilitation need.

##### Poor patient-PCPs relationships

Descriptions provided by the patients demonstrated the power imbalances that existed between the patients and their PCPs, with the patients proving to be mostly disadvantaged. For example, one elderly woman complained about the demeaning and disrespectful behavior of PCPs. Her experience of receiving conflicting instructions from different PCPs, with apparent disregard for her comfort, did not align with her expectation of receiving compassionate *“tender care”* (P10, rural PHC facility). Particularly in rural communities, younger ones are expected to ascribe respect to the matriarchs regardless of their socio-economic status. The power-imbalances minimize patients’ ability to make demands regarding the quality of care received from the PCPs. As a result, patients may shy away from actively participating in their treatment decisions including seeking required rehabilitation. This was shown in how another patient felt about their communication with PCPs.

“Sometimes I get scared, even though it's something I think of on my way here. But when I get to the nurses, I just don't say it. I go home… I worry that sometimes they would ask me too many questions that I would struggle to answer.” (P17, rural PHC facility).

The above expression indicates how patients have limited knowledge about their functioning problems and yet fear obtaining this knowledge from the PCPs who are supposed to assist them.

Patients distrust of PCPs also deterred patients from freely reporting their functioning problems as expressed by one participant from the rural areas who felt that the PCPs “*would tell other people.”* (P12, rural PHC facility) This is especially noteworthy in rural communities which are typically close-knit. The patients needed reassurance that the PCPs are trustworthy and value patient confidentiality for them to confide about their vulnerable status.

Past experiences of unmet healthcare needs further fueled patients’ distrust in the PCPs’ ability to help them. One participant reported being *“tired of singing the same chorus all the time, without receiving any help.”* (P23, traditional PHC facility). Thus, they were reluctant to report their functioning problems again.

##### Patients’ mental health challenges

Some participants had depressive disorders and anxiety associated with their health conditions which caused forgetfulness among other symptoms. This was expressed by one participant who explained that she did not report her functioning problems because *“sometimes [her] mind [was] not working so well”* (P21, traditional PHC facility). Mental health challenges due to their depressive state may limit patients’ ability to recall their functioning problems during consultation with the PCPs.

##### Limited consultation times

Another factor that affected patients’ ability to report their functioning problems was the limited consultation times which were caused by the clinics being busy, and either the PCPs or the patients arriving late for their appointments. One participant who explained why she arrived late for her appointments said the following:

“I get here late because I live so far, so by the time I get here, I just want to get the tablets which I came for and leave immediately so that I can get home on time. So sometimes I think if I report these things, they will take so much of my time that I would delay getting home.” (P11, rural PHC facility).

Patients may arrive on time for their appointments but due to busy clinics, they may not be attended to timeously. One participant described the effect of the long waiting times on the day of the consultation on reporting her functioning problems:

“No, I've never reported to the doctors. and that is because sometimes when we come to see nurses or doctors, they take a long time to come help us. So sometimes by the time you see the nurse you feel hungry, and you've already forgotten about other things you wanted to report.” (P10, rural PHC facility).

The hurried consultations led to poor communication and engagement between the patients and PCPs. As expressed above, it was often the case that the frustrated patients would rather compromise on reporting their functioning problems in favor of obtaining their chronic medications. The PCPs also ended up not taking enough time to conduct thorough medical reviews and effectively communicate with the patients. As a result, they may have failed to discern what was really concerning to the patients.

A few facilitators to reporting functioning problems during the PHC visit were mentioned.

##### Patients’ fear of dependency on others

Participants reported fearing continuous dependency on carers for carrying out their day-to-day function as one participant said.

“I just get very frustrated myself when I have to rely on people, that’s it because when you go from independent to having to be dependent on people, it’s a major shift around your whole lifestyle.” (P24, urban PHC facility).

Their loss of autonomy in deciding and controlling how and when they did things for themselves motivated patients to seek rehabilitation to restore optimal function. Embarrassment experienced by patients when the roles had shifted from being providers and decision-makers to asking for help and being left out of decisions may have reinforced the patients’ decision to seek rehabilitation.

##### Caregiver/family support during consultations

The presence of family particularly in attending consultations facilitated patients’ reporting of functioning problems to the PCPs. This was particularly so for patients with speech and cognitive functioning problems. Family members helped to facilitate communication between the patient and PCPs and clarify the patients’ concerns. One elderly patient reported how her *“daughter does report everything because she's the one who looks after [her]* (P21, traditional PHC facility). Thus, family played the additional role of being advocates for the patients, ensuring that the patients’ rehabilitation needs were met.

#### Referral to rehabilitation at PHC

The second category arising from the participants’ experiences at PHC related to referral to rehabilitation at PHC. Most patients reported not being referred to rehabilitation. As a result, patients remained uninformed regarding their need for rehabilitation or where to seek rehabilitation services. Six participants mentioned being referred to rehabilitation, most citing physiotherapy referral and one mentioning referral to a social worker for psychosocial rehabilitation. The doctor was the only cadre quoted as referring to rehabilitation.

##### Patients expected PCPs to be the stewards of their healthcare

Some participants left their healthcare decision-making to the PCPs and expected referral to rehabilitation to be solely initiated by the primary care provider. Thus, if the PCPs overlooked their need for rehabilitation, they may have concluded that rehabilitation was not necessary.

“The people who are responsible for that are the same people I report to when I get here. They are the ones who should be referring me to rehabilitation… It should be the nurses who come with a plan on how I should go about that process.” (P15, rural PHC facility).

##### Lack of patient empowerment

The converse was also true. Some patients’ expressions revealed the lack of a participatory approach in involving patients in their treatment decisions. Patients often felt that their voices were unheard while treatment decisions were made by the PCPs regardless of whether it was effective or not.

Because normally the doctors are just asking what you’re there for and that is it basically. You come to see them for whatever reason that you’re needing to see them and that’s basically it. (P24, urban PHC facility).

##### PCPs perceptions of patients’ functioning problems

PCPs held their own perceptions and stigmas towards functioning problems experienced by the patient. For example, the PCPs may undermine the severity or impact of the functioning problems.

“I first went to clinic, and they gave me all that stuff, that lady, she said no, it’s stress. I said no, it can’t be stress because it is painful and there is nothing that’s stressing me… She gave me a bottle of pills; she gave me some other pain killers and it didn’t help me.” (P25, urban PHC facility).

No, they have never referred me because it has never been something serious. (P20, traditional PHC facility).

Because the patient was unempowered, this implies that PCPs perceptions took precedence in determining the patients’ care including need for referral to rehabilitation.

##### Lack of rehabilitation services at PHC

Except for one, participants who reported being referred to rehabilitation were from PHC facilities that rendered rehabilitation services. This suggests a better awareness of rehabilitation among PCPs serving where rehabilitation services were provided than where rehabilitation was absent.

### Outcomes of PHC visit.

Though all the patients interviewed had functioning problems that were amenable to rehabilitation, only a few received rehabilitation at the PHC facilities. Others who had been referred to rehabilitation had to obtain it from higher levels of healthcare such as district hospitals.

In most cases, patients exited the PHC system without receiving any rehabilitation, perhaps obtaining medications which may have helped temporarily or not helped at all. One participant explained the reasons for not receiving rehabilitation as follows:

“I did not know that [I needed rehabilitation]. The other thing is that I do not know where to get them. Because I come to the hospital, and no one tells me to go to them.” (P16, rural PHC facility).

This suggests that by the time the patients leave PHC, a whole complex nexus of factors would have affected the patients’ ability to receive rehabilitation. Thus, many returned home without the needed support and information on how to cope with their functioning problems. Because the patients did not perceive the need for rehabilitation, the patients developed various coping strategies to accomplish the day-to-day activities expected of them. The reported coping strategies included acceptance, resilience, apathy, dependency on carers or use of self-prescribed medications.

### Patients’ perceptions of current primary care rehabilitation

The few participants who had experienced rehabilitation recounted how they perceived the quality of current rehabilitation services as well as factors which affected their re-access to rehabilitation at primary care. The themes, subthemes and categories related arising from their responses are presented in Table 4.

**Table 4:** Theme, subthemes, and categories related to experiences of rehabilitation services received at primary care

Theme	Subtheme	Category	Exemplary Quotation
Perceptions of current primary care rehabilitation service delivery	Low satisfaction with rehabilitation service delivery at primary care	Lack of assistive devices	*…the old man has started to experience stroke two days ago, he does not have a wheelchair or walking sticks and I also struggle with my own leg. So sometimes I struggle to help him.* (P8, urban PHC facility)
			*My concern was things like crutches. I got this one from people. The one I had is broken… Yes, I did [request crutches]. And they told me that they do not have crutches for now.* (P6, urban PHC facility)
		Rehabilitation professionals late or unavailable for booked appointments	*I’m not even sure if I’m going to see this physio today, that’s the problem where I’m at currently…because I was supposed to see her at ten today and unfortunately, it’s way after 11 already.* (P24, urban PHC facility)
		Poor communication regarding appointments	*Sometimes you would travel to the clinic or wherever you meet the nurses [rehabilitation professionals], and you would be told that they are not coming on that particular day, though you have been promised they would come."* (P7, urban PHC facility)
		Long waiting times for available appointments for rehabilitation	*I came last month and then she had a cancellation, so that’s how she could squeeze me in to today… So today I’m not sure how long I’m going to wait to get another appointment.* (P24, urban PHC facility)
	Positive functional outcomes	Restored level of function and independence	*Yes, I do see the difference and the rehabilitation is helping because when I see people who are experiencing or have the same illness, I am having, I can tell the difference…I used to be forgetful, I would forget my bank pin. But as I would come here and get some help, I started to improve on my memory as well… I started to feel much better and there was an improvement. Then I moved out of my sister’s place to stay by myself.* (P6, urban PHC facility)

*P* Participant *PHC* Primary Health Care

The experiences suggest that there are challenges in the delivery of rehabilitation services at primary care which impacted on patients’ ability to receive reliable, timely and adequate care. These challenges were related to unavailability of mobility aids, poor appointment mechanisms and shortage rehabilitation workers.

### Suggestions from patients for enhancing current primary care rehabilitation

A few participants gave suggestions on how rehabilitation services may be improved at primary care (Table 5). The suggestions were related to patient empowerment, adequacy of rehabilitation workforce and quality of rehabilitation service delivery. It is worth noting that not all patients had suggestions for improvement.

**Table 5:** Suggestions from patients on how rehabilitation services at primary care may be improved

Suggestion	Exemplary quotation
Patient and community participation	
Open communication to improve PCPs-patient partnership	*When I come to the clinic to report every kind of pain I am experiencing.* (P10, rural PHC facility)
Community engagement through the dissemination of information to the community to increase awareness of available rehabilitation services	*Yes. If you do that, people will know, they will know that… there will be physiotherapists there. And anyone who has problems related to that will be assisted. Just like me, I will also spread the word.* (P10, rural PHC facility)
Patient voice through anonymous platforms	*I believe that there should be things like suggestion boxes in every clinic… The people who should bring change to each clinic should open those boxes. Sometimes it is not easy to tell the nurse or to ask for such information because you feel like you are telling them what to do or you are teaching them their job.* (P5, urban PHC facility)
Rehabilitation human resources	
Ensure availability of relief rehabilitation professionals to attend to patients who had booked appointments	*I suppose if physios should be on time or there should be a relief physio or something to that affect to attend to those patients that’s got appointments because now, I know for a fact that if I get another date, it’s going to be a very late date because of the big amount of people that they have to see unfortunately.* (P24, urban PHC facility)
Rehabilitation service delivery	
Adequate assessment and diagnosis of functioning problems	*I believe that since I pulled my muscles. I believe that there's supposed to be therapy to investigate the cause of the incident.* (P1, urban PHC facility)
Improve patient education regarding self-management of functioning problems	*I do feel that there was some form of knowledge that I could have received to ease the pain.* (P4, urban PHC facility)
Improve patient education regarding available rehabilitation services	*You can just explain to the people who are waiting outside that there are going to be Physiotherapists here. If you do that, they will know.* (P10, rural PHC facility)
Provide telephonic communication with patients	*Something that I wish can be done is to have communication with the clinic. Maybe over the phone.* (P7, urban PHC facility)
Provide vocational training/support programs to address socio-economic integration	*There was a social worker here who I used to speak to, so we ended up being part of a project where we worked on one of the gardens. But I moved to the rural areas, so I think now it would be better if I can do that maybe once or to do something where I live…* (P7, urban PHC facility)
	*It would be great if the hospital could also assist with the social grant or food parcels because food is also scarce for him*. (P8, urban PHC facility)

*P* Participant *PHC* Primary Health Care

Additionally, patients stressed the importance of adequate assessment and diagnosis of functioning problems, patient education, and the provision of vocational training and support programs to address socio-economic integration.

## Discussion

According to our knowledge, this is the first study to describe patients’ journeys in accessing rehabilitation at primary care. Patients attending primary care in rural and urban South Africa reported several barriers and few facilitators that affected their access to and utilization of quality rehabilitation services at primary care. Although the patients had demonstrated their capability to overcome physical barriers by having already accessed PHC facilities, another layer existed deterring these patients from receiving essential rehabilitation care at this level of care. Additionally, a few recognizable differences in the patterns or factors affecting health seeking and utilization were noted between rural and urban participants. Few suggestions of how rehabilitation services at the primary care level may be enhanced were set forth.

The study highlighted the lack of standardized pathways for patients to access rehabilitation services at PHC, a barrier that has been previously reported in similar settings [35, 36]. None of the patients accessed rehabilitation services at primary care directly, if not re-accessing for follow-up appointments. Most patients required referral from the PCPs and even then, most needed to seek rehabilitation at higher levels of healthcare. This may be because rehabilitation professionals in the South African public healthcare sector are not typically considered first contact professionals [37]. In PHC settings, general practitioners, nurses, and community health workers are the initial point of contact for patients seeking medical attention for a particular health concern [38]. As found in our study, these first contact professionals may not adequately refer the patients to rehabilitation, which may be due to, among other reasons cited in the literature, poor awareness of the range of rehabilitation services or health conditions that can benefit from rehabilitation, absence of clear guidelines and protocols for rehabilitation referral, time constraints and fragmented healthcare systems [36, 38, 39]. In alignment with South Africa’s National Rehabilitation Policy and FSDR, there is need for intersectoral collaboration to enable provision of all components of rehabilitation – medical, psychosocial, educational, vocational and provision of assistive devices [11, 12]. It may be necessary to develop standardized referral pathways for rehabilitation services at PHC or consider the various care pathways to ensure patients receive equitable healthcare within South Africa's NHI [40].

The study highlighted several personal and contextual factors that resulted in the patients having a low perceived need for rehabilitation that potentially lowered patients’ demand for rehabilitation services at primary care. This finding contrasts Roberge et al. (2016) where patients recognized their need for mental health related rehabilitation because of the significant involvement of psychologists and social workers at primary care level [41]. In our study, though most patients were aware of their functioning problems, most were unaware of the benefits of rehabilitation or underestimated the severity of their functioning problems. Therefore, they could not prompt or make demands from rehabilitation services. Additionally, traditional healing practices, often ingrained in the cultural beliefs of rural communities, offered an acceptable alternative source of treatment where formal healthcare services were relatively less accessible or affordable [42]. According to Levesque's conceptual framework, healthcare utilization is an important proxy indicator of the accessibility of a healthcare system [17]. Efforts to invest in strengthening rehabilitation services at primary care may be futile if the end-users will not value or see the need to utilize them. Rehabilitation services at primary care will remain underutilized even after efforts have been made to make them available. Often, the PCPs are blamed for not referring adequately to rehabilitation. Merely educating PCPs on the value of rehabilitation may not be sufficient, as patient factors also need to be addressed to improve referral rates. This could involve collaborating with traditional healers in patient education and creating referral pathways.

Primary health system challenges that presented barriers to accessing rehabilitation were numerous and multi-faceted. The barriers identified are mostly consistent with the system-level challenges affecting provision of rehabilitation services in South Africa that were identified by Morris et al. (2019), which affected leadership and governance, human resources, service delivery, healthcare financing, medical technology, and information and research [43]. Patients with rehabilitation needs require ongoing support and care from primary care, but inadequate workforce, training, and limited resources, compromises the quality of healthcare received. Communication and attitudinal barriers between patients and PCPs result in fragmented care and poor care coordination between services [44] and commonly results in patient dissatisfaction [45] and delays or hesitancy in seeking rehabilitation services. Another significant problem affecting the receipt of quality rehabilitation services was the lack of assistive mobility devices. This problem causes challenges for these populations, where mobility-related functioning problems impact their livelihood significantly [5]. In these poorer populations it may not be possible to source assistive devices, even from private. However, our findings contrast one South African study that reported ample supply of assistive devices at an urban CHC [20]. In line with the National Rehabilitation Policy, equitable access to assistive devices could be improved by ensuring that there is planning and financial allocation and efficient procurement systems for procurement of priority assistive devices within the PHC system, [11] backed by strong political commitment to operational budgets and plans. Leveraging on local workmanship for cost-effective production of basic assistive devices as reported in the white paper on NHI may be useful [46]. Addressing the health system challenges requires a holistic approach to patient care, which includes ensuring that rehabilitation services are integrated into PHC settings.

The current study found that it was difficult for patients in poorly resourced contexts to access rehabilitation at primary care due to the complex interplay of personal, contextual and health system challenges. For example, the effect of socio-economic factors affected patients’ willingness to take responsibility for their treatment, preferring to place their confidence in healthcare professionals. According to some studies, poor education and poverty are associated with poor health literacy, and result in patients’ increased tendency to accept health professionals’ authority without question [47]. Partnerships are required for shared decision-making in which treatment decisions are guided by patient values, preferences and circumstances [45]. Considering the high levels of unemployment in South Africa, highest in the Eastern Cape at 42.8%, [48] and opportunities for paid employment even less in rural areas and for people with chronic health conditions, [49] patients may be faced with a trade-off between restoring their function or remaining qualifiable for government social security assistance [50]. In the rural areas, people mostly depend on subsistence farming for their livelihood, but this means of income may be lost when their health conditions limit participation in this activity or affect employability. In fact, doctors have perceived the abuse of the Grant system, whereby patients boycott rehabilitation to maintain the severity of their disability [51]. Thus, it may be important for rehabilitation to engage other sectors including labor, education, and housing to address the socio-economic factors that not only create health inequalities but also negatively impact individuals’ functioning.

### Recommendations

Addressing the challenges identified in this study will require a multi-faceted approach. The following evidence-based recommendations seek to expand on the suggestions that were raised by the current study participants.

Patient empowerment is critical in achieving the goal of strengthening rehabilitation in primary care [52]. Within the National Rehabilitation Policy, patient participation is considered a critical component of strengthening rehabilitation [11]. This includes increasing patients' awareness of their functioning problems, collaborative decision-making regarding rehabilitation goals and interventions, and active engagement in their treatment even where rehabilitation professionals may be absent, for example, through self-management strategies. Patient awareness campaigns can be conducted regularly to promote contextualized rehabilitation-related educational messages effectively [53]. Patients will thus have better knowledge about the causes and treatment of their functioning problems and be better informed about where to get help. Self-management programs led by lay people or PCPs have been found to positively impact some outcomes such as pain, depression, and quality of life [54]. Patient empowerment may go as far as training patients themselves including persons with disabilities to serve as rehabilitation workers [55]. Crucially, patients should be involved, not only in the development of rehabilitation policy, but also in the monitoring and evaluation implementation – an aspect identified as lacking in the context of the FSDR [56].

Innovation of evidence-based rehabilitation awareness platforms or guidance tools may ensure that the patients get practical, contextually relevant, and reliable information, [57] to guide them in the prevention and self-management of functioning problems related to their health conditions. Therefore, patients and carers can continue to be engaged in developing these tools to improve implementation and adherence. Mobile technology, in particular, has been found to potentially minimize the barriers to quality health care, including inadequate rehabilitation professionals, geographical barriers, and barriers emanating from patients' traditional beliefs and negative attitudes toward seeking treatment [58]. Notwithstanding, barriers to implementing digital technologies in low-resource settings will need to be considered, including lack of reliable network connectivity, electricity and patients’ limited access to mobile devices and affordable data [59]. It will be essential to determine how these tools or platforms may be integrated into established clinical treatment pathways to improve health outcomes and quality of life.

Suggestions were made to ensure that rehabilitation services continue to be provided in the absence of the resident rehabilitation professionals by employing substitute therapists. While this may be a challenge in low-resource settings, upskilling and task-shifting strategies may be employed to either expand or change the roles of available PCPs, as was done for mental health care in South Africa [60]. The available PCPs can be trained in the core rehabilitation competencies through the established comprehensive South African primary care course [61]. Easy-to-use clinical decision tools may guide them to identify functioning problems, provide basic rehabilitation services or refer appropriately to rehabilitation specialists. Considering that the PCPs working in low-resource settings may be already overburdened with high workloads, training local community volunteers [62] or introducing a ‘lower level’ cadre of rehabilitation assistants or technicians who are trained within a shorter time, [63] compared to the conventional degree programs, may increase the rehabilitation workforce in low-resource settings.

To support task-shifting strategies, rehabilitation specialists based at community rehabilitation centers, tertiary, or secondary facilities may use telerehabilitation to supervise non-rehabilitation clinicians and provide direct care in complex cases [64]. Telehealth may be further utilized to provide training and regular assistance to PCPs through regular presentations or consultation meetings to impart skills that enable greater competence and autonomy in managing functioning problems [65]. This will address the challenges related to inadequate rehabilitation workforce at primary care and the resultant long waiting times as well as the lack of clinical support. These collaborations would greatly improve awareness among the PCPs, especially at PHC facilities where rehabilitation services are not rendered and rehabilitation seems 'out of sight, out of mind.' However, several barriers may need to be considered in primary care contexts including technological barriers (e.g., poor Internet connectivity, unavailability of technological devices), and patient barriers (e.g., digital literacy and financial barriers) among others [66].

### Recommendations for future research

The perspectives of PCPs in exploring the challenges of rehabilitation service delivery at primary care level and obtaining suggestions on what could be done to enhance rehabilitation at primary care will consolidate the current study findings. The PCPs may offer additional insights on issues that patients may not be informed about, such as health care systems, governance, and policy.

### Limitations

Findings from qualitative studies cannot be generalized but remain useful to similar contexts or populations. However, transferability was enhanced by using purposive sampling techniques to ensure a diverse population and providing detailed and accurate accounts of the population. The study was confined to two regions of the Eastern Cape. It is possible that other regions of the Eastern Cape or provinces of the country have different issues that require exploration.

The PI was limited in understanding isiXhosa language and culture. A translator fluent in isiXhosa and with rehabilitation background helped to overcome the barrier.

## Conclusion

Our study has shown that patients attending PHC are not guaranteed access to primary care rehabilitation by virtue of having entered the PHC system. Much work remains to be done to bridge the gap between patients’ rehabilitation needs and provision of accessible and quality rehabilitation services at primary care in the Eastern Cape. As South African PHC systems respond to the changing burden of disease, it is important to consider the patient perspectives on their health needs and suggestions for enhancing care. The study highlighted the need to address patient factors impacting referral rates to rehabilitation. Patients need increased knowledge and awareness of their functioning problems and their need for rehabilitation as well as empowerment to fully engage in their rehabilitation treatment and decision-making regardless of their psychological, social, and economic status. Lessons learned from this study will provide valuable information for developing and implementing tailored strategies to enhance access, delivery, and receipt of rehabilitation services for adults attending primary care.

### Supplementary Information

**Additional file 1. **Demographic Data and Health Information Collection Form (Adults at PHC).

**Additional file 2. **Interview guide – Adults at PHC.

## Data Availability

The datasets used and/or analysed during the current study are available from the corresponding author upon reasonable request.

## Abbreviations

CHCs: Community Health Centres
HIV: Human Immuno-deficiency Virus
LMICs: Low to Middle Income Countries
NCDs: Non-Communicable Diseases
NHI: National Health Insurance
P: Participant
PCPs: Primary Care Providers
PHC: Primary Health Care
TB: Tuberculosis
