# Supplementary material for: Bridging the chasm between patients’ needs and current rehabilitation care: perceptions of adults presenting for primary care in the Eastern Cape
Source: BMC Health Serv Res. 2024 Feb 5;24:166. doi: 10.1186/s12913-024-10564-5 (PMC10840242; doi:10.1186/s12913-024-10564-5)
Supplement: Supplementary file 1 — Additional file 1. Demographic Data and Health Information Collection Form (Adults at PHC). [file 12913_2024_10564_MOESM1_ESM.pdf]

# Additional file 1.pdf: Demographic Data and Health Information Collection Form (Adults at PHC)

Data collection instrument to capture patients' socio-demographic and relevant clinical history.

| **DEMOGRAPHIC INFORMATION** | | | |
|---|---|---|---|
| **Surname:** | **First name:** | **Title:** | **Respondent** (patient or caregiver)**:** |
| **Date of birth *(dd/mm/yy)*:** _ _/_ _/_ _ | | **Gender:** ☐ female ☐ male | |
| **Preferred language:** | | **Marital status:** ☐ married ☐ unmarried | |

| **Section 2: Contact Details** | |
|---|---|
| **Mobile phone (if any):** | **House phone (if any):** |
| **E-mail address (if any):** | |
| **Caregiver (name and contact information):**<br>***For patients only** | |

| **Section 3: Address** |
|---|
| **Name of City or Town where you live:** |
| **Name of Suburb or Area where you live:** |

| **Section 3: Education History** |
|---|
| **Highest education/academic level you have achieved:** (for example, standard or grade completed at school) |
| **Years of formal education:** |

| **Section 4: Employment & Finances** | | |
|---|---|---|
| **Are you currently employed?** | ☐ Yes | ☐ No |
| **If YES, please describe your job/work:** | | |
| **If NO, please describe your previous job/work:** | | |
| **If NO, please tell us when you stopped working:** | | |
| **Source of Income now – check *all that apply:*** | | |
| ☐ Salary/Wages | ☐ Grants | ☐ Remittances (Gift) |
| ☐ Pension | ☐ Income from a business | ☐ Other (explain): ........................................................ |

| **Section 4: Medical Diagnosis of existing Main Health Conditions** |
|---|
| 1. No medical condition exists |
| 2. |
| 3. |
| 4. |
| 5. A health condition (disease, disorder, injury) exists; however, nature or diagnosis is not known |
| **BRIEF HEALTH INFORMATION:** |
| X1. Height: ________ cm |
| X2. Weight: ________ kg |
| X3. Dominant hand (prior to health condition): ☐ Left ☐ Right ☐ Both hands equally |
| X4. How do you rate your physical health in the past month? |
| ☐ Very good ☐ Good ☐ Moderate ☐ Bad ☐ Very bad |
| X5. How do you rate your mental and emotional health in the past month? |
| ☐ Very good ☐ Good ☐ Moderate ☐ Bad ☐ Very bad |
| X6. Do you currently have any disease(s) or disorder(s)? ☐ Yes ☐ No |
| *If YES, please specify:* |
| X7. Did you ever have any significant injuries that had an impact on your level of functioning? ☐ Yes ☐ No |
| *If YES, please specify:* |
| X8. Have you been hospitalized in the last year? ☐ Yes ☐ No |
| *If YES, please specify reasons and for how long?* |
| X9. Are you taking any medication (either prescribed or over the counter)? ☐ Yes ☐ No |
| *If YES, please specify major medications?* |
| X10. Do you smoke? ☐ Yes ☐ No |
| X11. Do you consume alcohol or drugs? ☐ Yes ☐ No |
| X13. Do you use any assistive devices such as glasses, hearing aids, wheelchairs, etc.? ☐ Yes ☐ No |
| *If YES, please specify:* |
| X14. Do you have any person assisting you with your self-care, shopping, or other daily activities? ☐ Yes ☐ No |
| *If YES, please specify person and assistance they provide:* |
| X15. Are you receiving any kind of treatment for your health? ☐ Yes ☐ No |
| *If YES, please specify:* |
| X15. Have you received any rehabilitation for your health? ☐ Yes ☐ No |
| *If YES, please specify:* |

| |
|---|
| |
| X16. Additional significant information on your past and present health: |
| X17. IN THE PAST MONTH, have you been totally unable to carry out your usual activities or work because of your health condition? (a disease, injury, emotional reasons or alcohol or drug use) ☐ Yes ☐ No |
| *If YES, how many days?* |
| X18. IN THE PAST MONTH, have you been totally unable to carry out your usual activities or work because of your health condition? (a disease, injury, emotional reasons or alcohol or drug use) ☐ Yes ☐ No |
| *If YES, how many days?* |

| If any of the following apply to you, please tick the box | |
|---|---|
| ☐ D1 Visual impairment | ☐ D8 Mental health issues e.g., worrying, feeling sad |
| ☐ D2 Communication problems e.g., talking and hearing | ☐ D9 Difficulty with self-care e.g., dressing, bathing, eating |
| ☐ D3 Mobility problems | ☐ D10 Difficulty with performing household chores |
| ☐ D4 Muscle function problems | ☐ D11 Weight problems |
| ☐ D5 Joint function problems | ☐ D12 Sexual problems |
| ☐ D6 Problems with your chest or breathing | ☐ D13 Learning/ work difficulty |
| ☐ D7 Emotional /behavioral difficulties | ☐ D14 Pain in any part of your body |
| ☐ Other disability, *please specify*: | |
| ***For administrative use only:*** | ***Questionnaire number: APHC-____*** |
