# Supplementary material for: Bridging the chasm between patients’ needs and current rehabilitation care: perceptions of adults presenting for primary care in the Eastern Cape
Source: BMC Health Serv Res. 2024 Feb 5;24:166. doi: 10.1186/s12913-024-10564-5 (PMC10840242; doi:10.1186/s12913-024-10564-5)
Supplement: Supplementary file 2 — Additional file 2. Interview guide – Adults at PHC. [file 12913_2024_10564_MOESM2_ESM.pdf]

Additional file 2.pdf Interview guide – Adults at PHC.

Data collection instrument guiding semi-structured interviews with participants.

(Focus of interview: To explore the impact of functioning problems on the life roles of adults attending PHC and suggestions on improving primary care rehabilitation in SA)

Please help me better understand your experience of problems with doing what matters most to you in daily life as well as the rehabilitation services you have sought.

Icebreaker: Tell us about your experiences: what do you enjoy doing, what are you good at?

Introductory question: Is there something you used to enjoy doing before that you cannot do now or that you must do differently?

What did you struggle or need help with after experiencing (name health condition)?

Back-up Q: How did your walking or ability to move around change? Why?

Back-up Q: How did your self-care tasks, like feeding, bathing, and dressing, change? Why?

Back-up Q: How did your thinking or memory, change? Why?

Back-up Q: How did your talking change? Why?

Back-up Q: How did your mood or emotions change? Why?

Key questions: If you think about your body, what functioning problems are most concerning to you? Why?

If you think about your daily life (at home, at work), which challenges in daily functioning are most concerning? Why? What is important to you that you cannot do now?

Back up Q: How was your life at home different after (name health condition)? Why?

Back up Q: How was your life at work different after (name health condition)? Why?

Back up Q: How was your income different after (name health condition)? Why?

How have your functioning problems affected the way you live in your community?

When you go to the clinic/community health centre regarding your functioning problems, what concerns you the most?

What have you expected the primary health care workers to assist you with?

What would you like rehabilitation workers specifically to help you do again?

In your opinion, what has prevented effective rehabilitation of your functioning problems?

Thinking of your overall experience with (name health condition) and the treatment you have had so far, please share any suggestions that could make rehabilitation care better.

What are your thoughts on using technologies like a smartphone or tablet to get information to guide you in managing your functioning needs? What concerns do you have? What would work better for you?

What would you like to know about how to manage your functioning problems?

Ending question: Is there anything else you which I haven't asked you which you would like to share with me?

Closure of Interview

Thank you for your time and for sharing your experiences with us.

I will stop the recording now.

My reflections on the interview process:
